# Fall in new HIV diagnoses among men who have sex with men (MSM) at selected London sexual health clinics since early 2015: testing or treatment or pre-exposure prophylaxis (PrEP)?

**DOI:** 10.2807/1560-7917.ES.2017.22.25.30553

**Published:** 2017-06-22

**Authors:** Alison E Brown, Hamish Mohammed, Dana Ogaz, Peter D Kirwan, Mandy Yung, Sophie G Nash, Martina Furegato, Gwenda Hughes, Nicky Connor, Valerie C Delpech, O Noel Gill

**Affiliations:** 1 HIV & STI Department, Centre for Infectious Disease Surveillance and Control (CIDSC), Public Health England, London, United Kingdom; 2 These authors contributed equally to this work and share first authorship

**Keywords:** HIV, diagnoses, testing, treatment, PrEP, Men who have sex with men, Treatment-as-Prevention London, England,

## Abstract

Since October 2015 up to September 2016, HIV diagnoses fell by 32% compared with October 2014–September 2015 among men who have sex with men (MSM) attending selected London sexual health clinics. This coincided with high HIV testing volumes and rapid initiation of treatment on diagnosis. The fall was most apparent in new HIV testers. Intensified testing of high-risk populations, combined with immediately received anti-retroviral therapy and a pre-exposure prophylaxis (PrEP) programme, may make elimination of HIV achievable.

Gay, bisexual and other men who have sex with men (MSM) account for half of all people living with HIV in England and are the group most at risk of acquiring HIV [1]. By end 2015, 94% (34,439/37,590) of MSM diagnosed with HIV in England received anti-retroviral therapy (ART), of whom 95% had supressed viral load (viral load < 200 mL) [1]. An additional 5,000–8,000 MSM were estimated to have undiagnosed infection [1-3].

Since 2012, national guidelines have recommended up to 3-monthly HIV testing for MSM at high risk of acquiring HIV [4,5] and starting ART regardless of CD4 count to prevent onward transmission (‘treatment as prevention’) [6,7]. Consequently, the number of men starting ART rose from 2,700 in 2013 to 3,600 in 2015 [1]. Beginning in 2013, pre-exposure prophylaxis (PrEP) has been available to some MSM as part of the ‘Pre-exposure prophylaxis to prevent the acquisition of HIV-1 infection (PROUD)’ trial [8] and more recently through international purchasing online [9,10]. In December 2016, selected London sexual health clinics reported a fall in HIV diagnoses among MSM [11]. A rapid analysis of surveillance and monitoring data was conducted to confirm and explain this fall.

## Data sources and analysis

Quarterly data from the genitourinary medicine clinic activity dataset (GUMCADv2) for January 2013–September 2016 [12] were used to examine HIV diagnoses and testing patterns among MSM attending one of the over 200 free, confidential, open-access sexual health clinics in England. Clinics that reported a large fall in diagnoses in the most recent year for which data were available, i.e. clinics with a > 20% decline and > 40 cumulative new HIV diagnoses between October 2014–September 2015 and October 2015–September 2016, were compared with other clinics in London and outside London. The number of HIV-negative MSM attending with a history of an HIV test and a bacterial sexually transmitted infection (STI) (> 90% were genital or rectal infections) was used as an indicator for those at high risk of HIV acquisition.

The HIV and AIDS Reporting System (HARS) [13] data, geographically aligned for clinics, for the most recent years (2013–2015) were used to examine: (i) trends in CD4 count within 91 days of HIV diagnosis; (ii) the number of MSM diagnosed with HIV who are untreated or treated but whose viral load is not suppressed; and (iii) time from HIV diagnosis to ART initiation. National estimates of HIV prevalence were stratified by the proportion of diagnosed and undiagnosed infection [3]. Estimates of the proportion of MSM with undiagnosed HIV infection [1] were calculated using the number of MSM with diagnosed infection to estimate the number of those undiagnosed in the catchment area of each clinic group.

### Fall in HIV diagnoses among men who have sex with men

**Figure 1:**
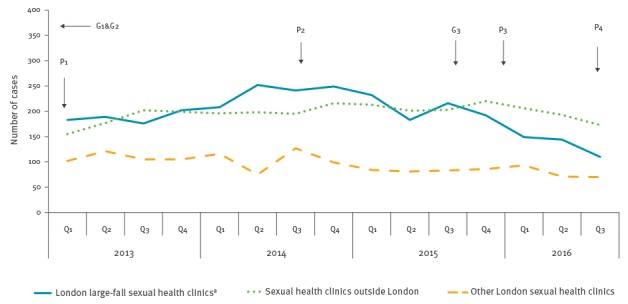
New HIV diagnoses among men who have sex with men attending sexual health clinics by year and quarter, England, 2013–2016 (n = 7,291 HIV diagnoses)

BASHH: British Association for Sexual Health and HIV; BHIVA: British HIV Association; MSM: men who have sex with men; PrEP: pre-exposure prophylaxis; PROUD: Pre-exposure prophylaxis to prevent the acquisition of HIV-1 infection; Q = quarter.

^a^ Defined as clinics with at least a 20% decrease in HIV diagnoses and over 40 cumulative diagnoses between October 2014–September 2015 and October 2015–September 2016.

**Figure 2:**
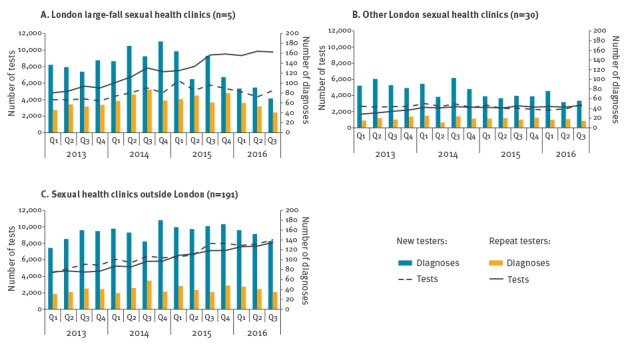
Number of HIV tests and diagnoses in men who have sex with men at sexual health clinics by new and repeat tests and clinic group, England, 2013–2016

Between October 2014–September 2015 and October 2015–September 2016, reported new HIV diagnoses among MSM fell by 17% (from 2,060 to 1,707) in England and by 25% (from 1,227 to 915) in London. Nationally, diagnoses among heterosexuals remained stable at 1,500 in both periods. A 32% decline was observed among five London large-fall clinics (from 880 to 595; p = 0.014 for test of linear trend in diagnoses by quarter) compared with 8% at 30 other London clinics (from 347 to 320, p = 0.115) and 5% (from 833 to 792, p = 0.101) in 191 clinics in the rest of England (Figure 1,2).

P1: Recruitment to the PROUD trial began with 50% of participants allocated to the deferred arm (12 months after trial commenced) [8].  P2: All deferred participants offered PrEP.  P3: Online purchasing of PrEP began in late 2015.  P4: Online purchasing of PrEP routine. Interpolating from the trial enrolment figures over time [5], an estimated 200 MSM were taking PrEP by end 2013, 500 by end 2014, and it is likely an additional few hundred by end 2016 with the majority of the latter via online purchase [9,10,14,15].

G1: In May 2012 BHIVA/ BASHH and in November 2012, Public Health England (at the time, the Health Protection Agency) recommended that high-risk MSM have an HIV test annually and up to every 3 months if having condomless sex with new or casual partners.

G2: In May 2012, BHIVA guidelines advised people could start ART with a CD4 count > 350 cells after discussion with the clinician should the patient wish to protect partners from sexual HIV transmission.

G3: In September 2015, BHIVA guidelines were strengthened to recommend treatment for all individuals regardless of CD4 count for the purpose of treatment as prevention.

Q: quarter.

### Changing patterns of HIV testing

Testing patterns were analysed from January 2013–September 2016. Among the large-fall clinics (Figure 2a), the number of HIV tests in MSM increased by 50% (from 8,820 in January–March 2013 to 14,820 in July–September 2016); the number of new testers, i.e. those not tested in the previous 2 years, was stable at around 5,000 per quarter, whereas the number of repeat testers i.e. those who had an HIV test within the previous 2 years increased by 60%, from 4,800 to 9,760. The 3-year rise in testing in the large-fall clinics coincided with an initial increase in HIV diagnoses through 2014 in both new and repeat testers and in early 2015 the decline was observed, predominantly in new testers. In other London clinics, the number of new and repeat testers remained stable, and outside London, new and repeat testers increased equally, although there was no discernible effect on HIV diagnoses in either setting (Figure 2b and c).

Over the period, the number of MSM attending clinics increased by 4% for both groups of London clinics, and by 16% outside of London. Importantly, the volume of testing at the large-fall clinics was such that 41% (58,180/140,980) of HIV tests in MSM attending clinics in England during October 2015–September 2016 occurred at one of these five clinics. Exceptionally, the median CD4 count at HIV diagnosis of men diagnosed at large-fall clinics increased substantially (from 469 in 2013 to 548 in 2015). In contrast, the median CD4 count rose only from 442 to 489 in other London clinics, and remained around 430 outside London, over the same period. This indicates that the testing volumes and frequency of testing carried out in these settings were still insufficient to substantially reduce the average time from infection to diagnosis compared with large-fall clinics.

### Prompt treatment following HIV diagnosis

**Figure 3:**
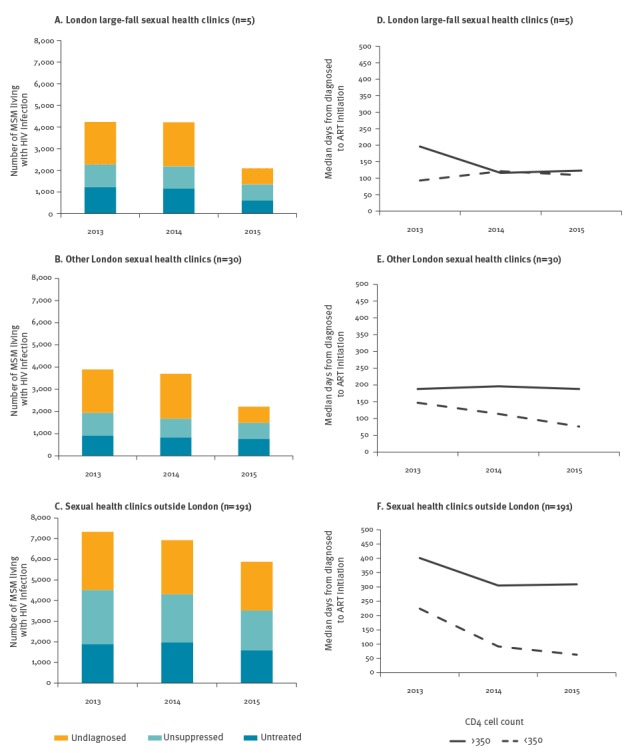
Numbers of men who have sex with men living with HIV infection who are undiagnosed, diagnosed and untreated or treated and non-supressed viral load (A-C) and median time (days) from HIV diagnosis to ART initiation, by CD4 count at ART start (D-F) by clinic group, England, 2013–2015

ART: anti-retroviral therapy; MSM: men who have sex with men.

Although the number of MSM living with diagnosed HIV infection who were untreated declined by 27% in England (from 4,025 in 2013 to 2,950 in 2015), this decline was greatest at large-fall clinics (51%; from 1,224 to 601) compared with other London clinics (17%; from 906 to 754) and clinics outside London (16%; from 1,895 to 1,595) (Figures 3a-c). Moreover, while there has been a general reduction in the time to starting ART in those with a CD4 count > 350 at onset of ART, the median time from diagnosis to treatment in 2015 was substantially shorter at large-fall clinics (120 days) compared with other London clinics (190 days) and clinics outside London (260 days) (Figures 3d-f).

Treatment data and viral load data are adjusted for missing information (99% and 84% respectively): CD4 cell count taken within 91 days of diagnosis, available for 91% (7,519/8,297) of records; CD4 count at ART initiation, available for 76% (6,960/9,150) of records. Year-specific estimates of proportion of all MSM with HIV who are undiagnosed were obtained using Multi-Parameter Evidence Synthesis [1,3].

### Men who have sex with men with transmissible levels of virus

MSM with transmissible levels of virus include those diagnosed who are untreated or treated with a viral load > 200 copies/mL, as well as those with an undiagnosed infection (Figure 3a-c). In 2015, there were an estimated 10,190 MSM with transmissible levels of virus in England: 29% (n = 2,950) untreated, 34% (n = 3,420) unsuppressed and 37% (n = 3,820) undiagnosed. In the same year, among clinic attendees, the ratio of MSM with transmissible levels of virus to MSM at high-risk of HIV acquisition was 0.6 (2,088/3,596) at large-fall clinics, 2.6 (2,219/868) at other London clinics and 2 (5,877/2,933) at clinics outside London. Assuming that sexual networks broadly correspond with clinic attendance patterns, the documented ratio differences suggest that MSM at high risk of HIV acquisition who attended one of the large-fall clinics have a much lower likelihood of exposure to a man with transmissible levels of virus.

### Availability of pre-exposure prophylaxis

Available data suggest the number of MSM who began PrEP in England either as trial participants or via online purchase has been limited to date. Although all five large-fall clinics participated in the PROUD trial, three other clinics in London and five clinics outside London did so as well. An estimated 200 MSM were taking PrEP by end 2013, 500 by end 2014 [8,14] and it is likely an additional few hundred by end 2016 [9,10,15].

Assuming a best prevention case scenario of a 9% annual HIV incidence, the very high-risk level as observed in the PROUD trial [8], by end 2015, the cumulative number of HIV infections directly prevented by PrEP would have been 90 at most. Not all of them would have attended large-fall clinics and of those who did, the decline in directly prevented infections would have been most apparent in repeat HIV testers.

## Limitations

Though powerful, the surveillance and monitoring data needs cautious interpretation, especially given the post-hoc nature of the analysis. Conclusions could be affected by reporting delay (albeit minimal), incomplete data in relation to ART coverage, ART start date and CD4 count at HIV diagnosis, and neither the impact of partner notification nor the movement between clinics for HIV testing is measured. The assumption that attendees of the same clinics are more likely to form part of the same sexual network compared with random sexual mixing is plausible but unsubstantiated. Finally, while numbers of HIV diagnoses are not synonymous with HIV incidence, the rise in median CD4 count at HIV diagnosis suggests that the fall in diagnoses reflects a fall in incidence.

## Conclusions

The 17% fall in new HIV diagnoses in MSM in England between October 2014–September 2015 and October 2015–September 2016 was focussed in five clinics which experienced a 32% decline. The fall seen at these five clinics coincided with accelerated treatment at diagnosis and a substantial increase in HIV testing, particularly repeat testing.

The volume of HIV tests across London combined with rapid treatment following diagnosis at the five large-fall clinics is now likely to have reached a level that decreases the number of men with transmissible levels of virus thereby reducing transmission. The use of PrEP among high-risk MSM, although limited at this stage, will also have contributed to the fall in new diagnoses. If HIV testing of MSM at high risk of HIV is intensified, and wide-scale immediate ART, as observed within the London large-fall clinics, is replicated elsewhere, it is probable that a substantial reduction in HIV transmission among MSM could be achieved nationally. Should the promise of the ‘PrEP Impact Trial’ proposed in England [16] be realised promptly, then a very large reduction in HIV transmission in MSM may be attained. The similarity of the MSM HIV epidemic in England to elsewhere in western Europe [17] suggests a similar approach in these countries might be equally successful.

## References

[1] Public Health England (PHE). HIV in the UK - 2016 report. London: PHE; Dec 2016. Available from: https://www.gov.uk/government/uploads/system/uploads/attachment_data/file/602942/HIV_in_the_UK_report.pdf

[2] Birrell PJ Gill ON Delpech VC Brown AE Desai S Chadborn TR HIV incidence in men who have sex with men in England and Wales 2001-10: a nationwide population study. Lancet Infect Dis. 2013;13(4):313-8. 10.1016/S1473-3099(12)70341-9 23375420 PMC3610092

[3] Goubar A Ades AE De Angelis D McGarrigle CA Mercer CH Tookey PA Estimates of human immunodeficiency virus prevalence and proportion diagnosed based on Bayesian multiparameter synthesis of surveillance data. J R Stat Soc Ser A Stat Soc. 2008;171(3):541-80. 10.1111/j.1467-985X.2007.00537.x

[4] Clutterbuck D, Flowers P, Barber T, Wilson H, Nelson M, Hedge B, et al. UK National Guidelines on safer sex advice. London: The Clinical Effectiveness Group of the British Association for Sexual Health and HIV (BASHH) and the British HIV Association (BHIVA); 2012. [Accessed 16 Apr 2017]. Available from: http://www.bhiva.org/documents/Guidelines/SaferSex/BASHH_BHIVA_Safer_Sex_Advice_WebFinal120712.pdf

[5] Health Protection Agency (HPA). HIV in the United Kingdom: 2012. London: HPA; Nov 2012 Available from: https://www.gov.uk/government/uploads/system/uploads/attachment_data/file/335452/HIV_annual_report_2012.pdf

[6] Williams I Churchill D Anderson J Boffito M Bower M Cairns G Writing Group British HIV Association guidelines for the treatment of HIV-1-positive adults with antiretroviral therapy 2012 (Updated November 2013. All changed text is cast in yellow highlight.). HIV Med. 2014;15(Suppl 1):1-85. 10.1111/hiv.12119 24330011

[7] British H. IV Association (BHIVA). British HIV Association guidelines for the treatment of HIV-1‐positive adults with antiretroviral therapy. London: BHIVA; 2015. Available from: http://www.bhiva.org/documents/Guidelines/Treatment/2015/2015-treatment-guidelines.pdf

[8] McCormack S Dunn DT Desai M Dolling DI Gafos M Gilson R Pre-exposure prophylaxis to prevent the acquisition of HIV-1 infection (PROUD): effectiveness results from the pilot phase of a pragmatic open-label randomised trial. Lancet. 2016;387(10013):53-60. 10.1016/S0140-6736(15)00056-2 26364263 PMC4700047

[9] Prepster. [Accessed 16 April 2017]. Available from: http://prepster.info/

[10] I want PrEP now. [Accessed 16 April 2017]. Available from: https://www.iwantprepnow.co.uk/#

[11] Cairns G. National AIDS Manual (NAM)-aidsmap. London: NAM; 22 Dec 2016. Available from: http://www.aidsmap.com/The-UKs-largest-sexual-health-clinic-saw-a-40-drop-in-new-HIV-infections-this-year/page/3106754/

[12] Savage EJ Mohammed H Leong G Duffell S Hughes G Improving surveillance of sexually transmitted infections using mandatory electronic clinical reporting: the genitourinary medicine clinic activity dataset, England, 2009 to 2013. Euro Surveill. 2014;19(48):20981. 10.2807/1560-7917.ES2014.19.48.20981 25496573

[13] Rice, BD, Yin Z, Brown AE, Croxford S, Conti S, De Angelia D, et al. Monitoring of the HIV Epidemic Using Routinely Collected Data: The Case of the United Kingdom. AIDS Behav. 2016; [Epub ahead of print]. PMID: 27832390. 10.1007/s10461-016-1604-6 27832390

[14] Dolling DI Desai M McOwan A Gilson R Clarke A Fisher M PROUD Study Group An analysis of baseline data from the PROUD study: an open-label randomised trial of pre-exposure prophylaxis. Trials. 2016;17(1):163. 10.1186/s13063-016-1286-4 27013513 PMC4806447

[15] Aloysius I, Zdravkov J, Whitlock G, Alldis J, Nwokolo N, Aylward A, et al. InterPrEP (II): internet-based pre-exposure prophylaxis (PrEP) with generic tenofovir DF/emtricitabine (TDF/FTC) in London: analysis of safety and outcomes. Abstract P32 presented at the 23rd Annual Conference of the British HIV Association (BHIVA) Liverpool, UK4-7 April 2017.

[16] Public Health England (PHE) / National Health Service (NHS) England. NHS England and PHE extend HIV prevention programme. PHE/NHS: London; 4 Dec 2016. Available from: https://www.gov.uk/government/news/nhs-england-and-phe-extend-hiv-prevention-programme <

[17] European Centre for Disease Prevention and Control (ECDC) / World Health Organization Regional Office for Europe (WHO/Europe). HIV/AIDS surveillance in Europe 2015. Stockholm: ECDC; 2016. Available from http://ecdc.europa.eu/en/publications/Publications/HIV-AIDS-surveillance-Europe-2015.pdf

